# Antibiotic Discontinuation 24 h After Neonatal Late-Onset Sepsis Work-Up—A Validated Decision Tree Model

**DOI:** 10.3389/fped.2021.693882

**Published:** 2021-08-19

**Authors:** Ori Goldberg, Nir Sokolover, Ruben Bromiker, Nofar Amitai, Gabriel Chodick, Oded Scheuerman, Haim Ben-Zvi, Gil Klinger

**Affiliations:** ^1^Neonatal Intensive Care Unit, Schneider Children's Medical Center of Israel, Petah Tikva, Israel; ^2^Sackler School of Medicine, Tel Aviv University, Tel Aviv, Israel; ^3^Division of Respiratory Medicine, Hospital for Sick Children, Toronto, ON, Canada; ^4^Department of Pediatrics B, Schneider Children's Medical Center of Israel, Petah Tikva, Israel; ^5^Maccabi Institute of Health Services Research, Maccabi Health Care Services, Tel Aviv, Israel; ^6^Infectious Disease Unit, Schneider Children's Medical Center of Israel, Petah Tikva, Israel; ^7^Microbiology Laboratory, Rabin Medical Center, Petah Tikva, Israel

**Keywords:** late-onset sepsis, neonate, antibiotic discontinuation, neutrophil-to-lymphocyte ratio, C-reactive protein, decision tree (CHAID)

## Abstract

**Objectives:** Neonatal late-onset sepsis work-up is a frequent occurrence in every neonatal department. Blood cultures are the diagnostic gold standard, however, a negative culture prior to 48–72 h is often considered insufficient to exclude sepsis. We aimed to develop a decision tree which would enable exclusion of late-onset sepsis within 24 h using clinical and laboratory variables.

**Study Design:** Infants evaluated for late-onset sepsis during the years 2016–2019, without major malformations, in a tertiary neonatal center were eligible for inclusion. Blood cultures and clinical and laboratory data were extracted at 0 and 24 h after sepsis work-up. Infants with bacteriologically confirmed late-onset sepsis were compared to matched control infants. Univariate logistic regression identified potential risk factors. A decision tree based on Chi-square automatic interaction detection methodology was developed and validated.

**Results:** The study cohort was divided to a development cohort (105 patients) and a validation cohort (60 patients). At 24 h after initial evaluation, the best variables to identify sepsis were C-reactive protein > 0.75 mg/dl, neutrophil-to-lymphocyte ratio > 1.5 and sick-appearance at 24 h. Use of these 3 variables together with blood culture status at 24 h, enabled identification of all infants that eventually developed sepsis through the decision tree model. Our decision tree has an area under the receiver operating characteristic curve of 0.94 (95% CI: 0.90–0.98).

**Conclusions:** In non-sick appearing infants with a negative blood culture at 24 h and normal laboratory values, sepsis is highly unlikely and discontinuing antibiotics after 24 h is a viable option.

## Background

Neonatal late-onset sepsis (LOS) is an important worldwide health care issue commonly occurring in Neonatal Intensive Care Units (NICU's) (1–3). Exclusion of neonatal sepsis is challenging as symptoms are often non-specific, diagnostic tests lack sufficient sensitivity and specificity and blood cultures, which are the diagnostic gold standard, often require 48–72 h until becoming positive in septic infants. As a result, infants suspected of having sepsis often receive antibiotics for 48–72 h until sepsis is excluded.

Many infants at risk of LOS are treated with antibiotics as the consequences of untreated sepsis are severe compared to the side effects of short-term antibiotic therapy (2). Thus, physicians often prefer to err on the side of caution and to continue antibiotics until there is virtually no risk of sepsis. The direct result is increased hospital length of stay, increased health care costs, increased antibiotic use and increased development of resistance to antibiotics. Short-term effects have been shown in preterm infants and result in an increase in adverse outcomes such as necrotizing enterocolitis and sepsis (4). However, long-term effects may be more important and should be considered such as the effect of antibiotics on infants' gut microbiota (5) and subsequent altered risk for obesity, asthma and allergies (6, 7).

Markers of neonatal LOS such as clinical signs and assessments, C-reactive protein (CRP), procalcitonin or complete blood count (CBC) are used for diagnosis of LOS, however none have shown sufficient diagnostic accuracy to be used as a single marker for early and accurate diagnosis of LOS (8–10), or even more so for exclusion of LOS. Our aim was to create a decision tree using clinical findings and laboratory tests that can be used to safely exclude sepsis in low-risk infants within 24 h after initial evaluation.

## Methods

### Study Population and Data Collection

Our retrospective case-control study was conducted in the NICU of Schneider Children's Medical Center of Israel. The NICU is a university-affiliated tertiary care pediatric medical center, which admits 900 newborns annually and serves about 9000 deliveries per year. The study was approved by the local Research Ethics Board (Study number RMC-18-142).

The NICU database routinely collects data on all admitted infants. A retrospective search of the database was performed for all infants diagnosed with primary culture-proven LOS between January 2016 and December 2019. We validated the diagnosis of LOS by chart review and by comparison with all infants identified by the microbiology laboratory as having positive blood culture results meeting the definition of LOS. During the study period, repeated blood cultures were taken when clinical deterioration occurred or to ensure bacterial eradication when blood cultures were positive. If infants had multiple episodes of LOS, only the first episode was included. Only infants hospitalized in the NICU from birth were included. There was no corrected gestational age cutoff limit. Excluded were infants with any major congenital malformation or any episode of early-onset sepsis. A control group of infants evaluated tor sepsis with negative blood cultures was chosen matching for prematurity and corrected gestational week in a 1:2 ratio. Infants were categorized as either preterm (below 34 weeks gestation) or near-term to full-term (equal or above 34 weeks gestation).

Data were extracted from the medical files as follows: demographic data (gestational age, birth weight, and gender), hospitalization characteristics at sepsis work-up (presence of central line, parenteral nutrition, day of life at time of sepsis work-up, antibiotic treatment, diagnoses of sepsis or necrotizing enterocolitis). Initial and repeated clinical and laboratory data measurements were collected during the time period starting 6 h prior to sepsis work-up and until 24 h after work-up and included clinical data (temperature, blood pressure, apneic or bradycardic episodes, ventilation requirement) laboratory data [automated CBC, neutrophil to lymphocyte ratio (NLR), CRP, blood glucose] and microbiological data (blood culture result, time to positivity of blood culture). Immature to mature neutrophil ratio is not routinely used in our institution and was not assessed.

For every patient, a health evaluation score was assigned using a scoring sheet by two senior neonatologists, who blindly evaluated the health record at time of sepsis work-up and 24 h after work-up. Disagreement between evaluating neonatologists was resolved by discussion or consulting a third neonatologist until consensus was achieved. The heath evaluation score (Appendix 1) was graded into three groups as follows: healthy, equivocal, or sick appearing (11) and is similar to the classification of clinical signs used routinely to evaluate early-onset sepsis (12, 13).

### Definitions

The study definitions have been previously described in detail (11). LOS was defined by a single positive blood culture by a causative pathogen 72 h or later after birth, with the exception of sepsis caused by coagulase negative staphylococci, which required two positive blood cultures. Each isolate was identified using the VITEK 2 system (bioMérieux, SA, France) and/or MALDI Biotyper System (Bruker Daltonics, Bremen, Germany), in accordance with the manufacturers' instructions for bacterial identification. Temperature abnormality was defined as below 36°C or above 37.5°C. Tachycardia was defined as a heart rate above 186 (bpm) (14). Hyperglycemia was defined as a glucose value above 108 mg/dl (14). CBC abnormalities (15–18) were defined as follows: leukocyte count below 5,000/mm^3^ or above 20,000/mm^3^, neutrophil count below 1,800/mm^3^ or above 5,400/mm^3^. Lymphopenia (18) was defined as lymphocyte count below 2,000/mm^3^. Thrombocytopenia was defined as a thrombocyte count below 150,000/mm^3^. Abnormal CRP (>0.75 mg/dl) and abnormal NLR (>1.5) were identified and calculated using receiver operating characteristic (ROC) curve, as described previously in a near identical cohort (11).

### Statistical Analysis

The entire study cohort was divided into a development cohort (about two-thirds of the entire cohort) and a validation cohort. The development cohort was used to build our model and decision tree and based on data from January 2016 through July 2017. The temporal validation cohort was used to evaluate our findings and based on data from August 2017 through December 2019. All data are expressed as mean and standard deviation (SD), median and interquartile range, or frequency and percentage. Unless otherwise indicated SD is presented in parenthesis. Categorical variables were compared using chi-square test or Fisher's exact test, as appropriate. Ordinal variables were compared using the Mann–Whitney test, and continuous variables were analyzed by the Student's *t*-test. Univariate logistic regression using variables available within 24 h after initial work-up was performed to identify factors associated with risk of LOS. Odds ratio (OR) and 95% confidence intervals (CI) were computed. A two-tailed *p*-value < 0.05 was accepted as statistically significant. A decision tree based on Chi-square automatic interaction detection methodology (19) was created using variables identified in the univariate analysis with a *p*-value < 0.2. Our decision tree performance was assessed by comparing the area under the curve (AUC) of the ROC curve of the development and temporal validation cohorts. Exclusion of LOS based on the decision tree was compared to previous predictors using AUC's, sensitivity, specificity, PPV and NPV, similar to previously described methodology (20).

Statistical analyses conformed to the Transparent Reporting of a multivariable prediction model for Individual Prognosis or Diagnosis (TRIPOD) criteria (21). All statistical analyses were performed using the IBM SPSS statistical package (SPSS 24, Chicago, IL, USA).

## Results

During the study period, culture positive LOS was identified in 140 of 3263 infants (4.3%) that were admitted to our NICU, representing 145 episodes. Multiple episodes of sepsis occurred in 3 infants. Of the 140 infants with culture positive sepsis, 50 were excluded because of major malformations, 36 due to a prior episode of early-onset sepsis and 1 patient due to missing data. The remaining 53 infants with LOS were matched to 112 control infants.

The entire study cohort was divided in a 2:1 ratio, to a development cohort (33 infants with LOS, 72 control infants) and a temporal validation cohort (20 infants with LOS, 40 control infants).

Gram-negative bacteria were the primary pathogens identified in 25 (47.2%) infants, followed by coagulase negative staphylococci found in 18 (34.0%) infants and other Gram-positive bacteria in 10 (18.9%) infants. At 24 h after LOS work-up, 21 of 53 (39.6%) of blood cultures where positive with a median time to positivity of 25.9 h (interquartile range 18.7–37.3 h). The demographic and clinical characteristics of the development and validation cohorts are shown in Table 1. There were no significant differences in the corrected mean gestation age and birth weight of the LOS vs. control groups, however, central line use was significantly higher in the LOS group. Comparison of clinical and laboratory predictors of infants with vs. without LOS within 24 h of sepsis work-up is presented in Table 2.

**Table 1:** Demographic and clinical characteristics of infants undergoing late-onset sepsis evaluation.

**Background characteristics**	**Training cohort (** ***n*** **=** **105)**				**Validation cohort (*n* = 60)**
	**All**	**No sepsis (*n* = 72)**	**Sepsis (*n* = 33)**	***P*-value**	
Mean corrected gestation at sepsis work-up (weeks) (SD^*^)	32.3 (3.3)	32.7 (2.6)	31.5 (4.7)	0.17	31.5 (3.3)
Gestational age <34 weeks (*n*) (%)	74 (70.5)	51 (70.8)	23 (69.7)	0.90	48 (80.0)
Female gender (*n*) (%)	51 (48.6)	36 (50.0)	15 (45.5)	0.67	26 (43.3)
Mean birthweight (gr) (SD)	1,440 (660)	1,484 (566)	1,343 (831)	0.34	1,387 (707)
Central line (*n*) (%)	28 (26.7)	12 (16.7)	16 (48.5)	<0.001	18 (30.8)
Parenteral nutrition (*n*) (%)	47 (44.8)	19 (26.4)	28 (84.4)	<0.001	27 (45.0)
Susp. Necrotizing enterocolitis (*n*) (%)	31 (29.5)	23 (31.9)	7 (21.2)	0.26	13 (21.7)

* *SD, standard deviation*.

**Table 2:** Comparison of clinical and laboratory predictors of infants with vs. without culture positive sepsis within 24 h of work-up.

**Cohort characteristics**	**Total (*n* = 105)**	**No sepsis (*n* = 72)**	**Sepsis (*n* = 33)**	Univariate Odds ratio (95% CI^**^)	***P*-value**
Health evaluation at 24 h^*^(*n*) (%)
Healthy	51 (48.6)	49 (68.1)	2 (6.1)	1	
Equivocal	38 (36.2)	21 (29.2)	17 (51.5)	19.8 (4.2–93.6)	0.001
Sick appearing	16 (15.2)	2 (2.8)	14 (42.4)	171.5 (22.1–1330.5)	<0.001
Median NLR^***^ ratio (IQR)	1.2 (0.6–4.5)	0.7 (0.6–1.4)	6.0 (3.4–3.5)	1.2 (1.1–1.35)	0.005
NLR (>1.5) (*n*) (%)	50 (47.6)	17 (23.6)	33 (100.0)	212.5 (12.4–3650.1)	<0.001
Median CRP^#^ (mg/dl) (IQR)	0.1 (0.0–3.9)	0.01 (0.0–0.2)	7.0 (3.6–10.0)	2.3 (1.7–3.2)	<0.001
CRP (>0.75 mg/dl) (*n*) (%)	40 (38.1)	10 (13.9)	30 (90.9)	62.0 (15.9–242.0)	<0.001
CRP (>0.75 mg/dl) or NLR (>1.5) (*n*) (%)	51 (48.6)	18 (25.0)	33 (100.0)	197.4 (11.5–3384.3)	<0.001
Platelets (<150,000mm^3^) (*n*) (%)	21 (20.2)	4 (5.6)	17 (51.5)	17.8 (5.2–60.2)	<0.001
Central line (*n*) (%)	28 (26.7)	12 (16.7)	16 (48.5)	4.7 (1.8–11.8)	0.002
Parenteral nutrition (*n*) (%)	47 (44.8)	19 (26.4)	28 (84.4)	15.6 (5.3–46.3)	<0.001
Tachycardia (maximal) (rate > 186) (%)	88 (85.4)	61 (87.1)	27 (81.8)	0.7 (0.2–21)	0.48
Abnormal temperature^##^ (*n*) (%)	30 (28.6)	20 (27.8)	10 (30.3)	1.1 (0.5–2.8)	0.79
Hyperglycemia^###^ (*n*) (%)	45 (42.9)	23 (31.9)	22 (66.7)	4.3 (1.7–10.2)	0.001
Abnormal white blood count (*n*) (%)^§^	27 (25.7)	13 (18.1)	14 (42.4)	3.3 (1.3–8.3)	0.008
Abnormal neutrophil count^§§^ (*n*) (%)	59 (56.2)	29 (40.3)	30 (90.9)	14.8 (4.1–53.2)	<0.001
Lymphopenia (minimal)^§§§^ (*n*) (%)	28 (26.7)	6 (8.3)	22 (66.7)	22.0 (7.3–66.5)	<0.001

* *Time from initiation of work-up;*

** *CI, confidence interval;*

*** *NLR, neutrophil to lymphocyte ratio;*

# *CRP, C-reactive protein;*

## *temperature <36 C or > 37.5 C;*

### *Glucose > 108 mg/dl; ^§^white cell count <5,000/mm^3^ or > 20,000/mm^3^; ^§§^neutrophil count <1,800/mm^3^ or > 5,400/mm^3^; ^§§§^lymphocyte count <2,000/mm^3^*.

The 3 single predictors for sepsis with the highest OR's were sick appearance (OR 171.5, 95% CI, 22.1–1,330.5), CRP > 0.75 mg/dl (OR 62.0, 95% CI, 15.9–242.0) and NLR > 1.5 (OR 212.5, 95% CI, 12.4–3,650.1). Fifty-seven percentage of infants with LOS did not appear sick at 24 h from work-up (in comparison to 97.8% of control infants). CRP and NLR continued to be robust predictors even when compared only to non-sick appearing control infants; for CRP > 0.75 mg/dl the OR was 36.2 (95% CI, 8.8–149.2) and for NLR > 1.5 the OR was 139.6 (95% CI, 8.0–2445.8).

**Figure 1:**
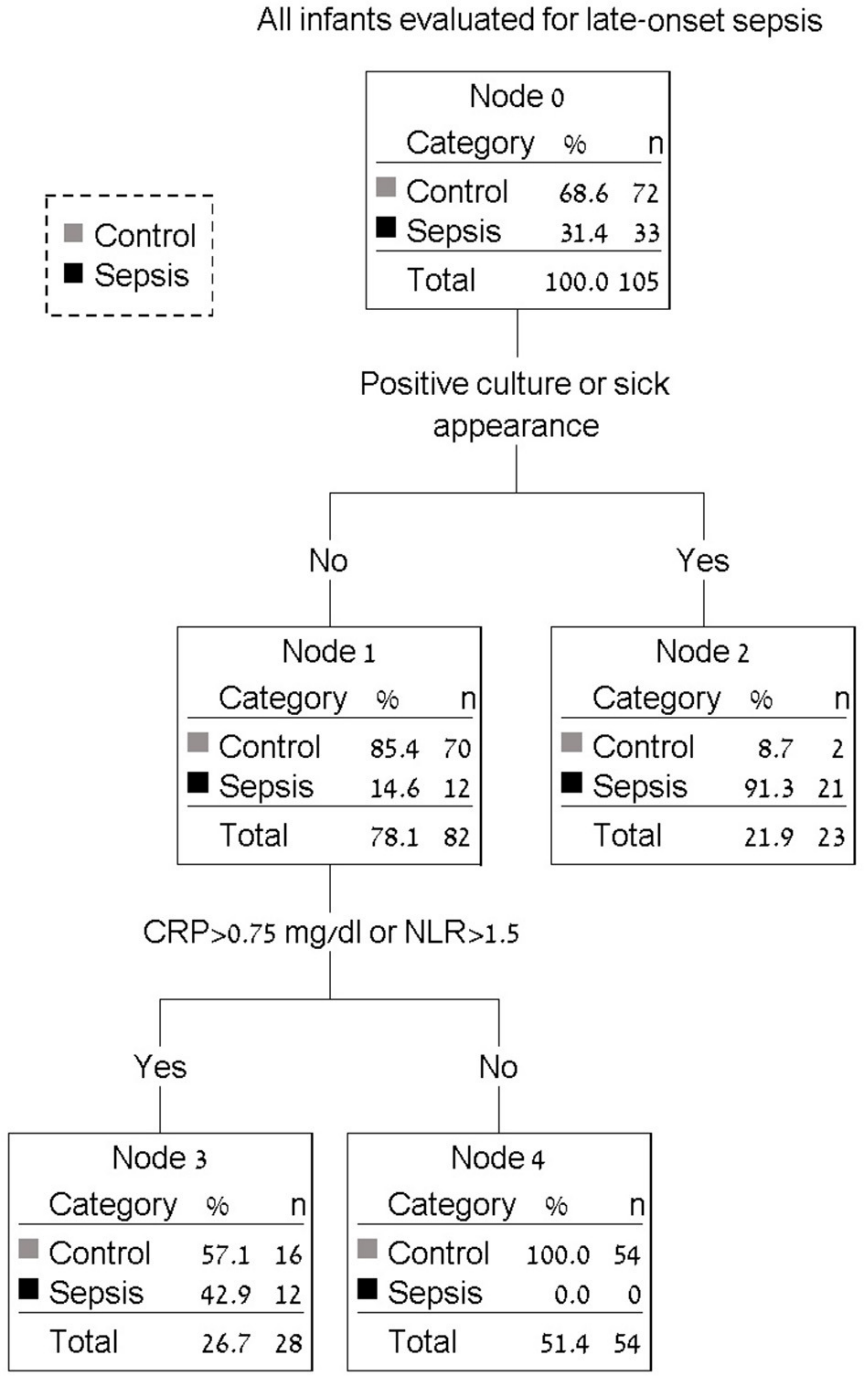
Decision tree for antibiotic discontinuation 24 h after neonatal late-onset sepsis work-up. All infants with positive blood culture or sick appearance will continue antibiotics beyond 24 h (node 2). Infants with elevated CRP (c-reactive protein) or elevated NLR (neutrophil to lymphocyte ratio) will also continue antibiotics beyond 24 h (node 3). Antibiotic therapy may be discontinued in all remaining infants (node 4).

Use of a decision tree enabled identification of all infants with LOS at the 24-h mark. The combination of blood culture, clinical assessment (sick appearance) with CRP > 0.75 mg/dl or NLR > 1.5 identified 33 of 33 infants with sepsis (100%) with only 18 of 72 (25.0%) control infants considered as possible sepsis beyond 24 h. Figure 1 presents a decision tree summarizing the approach to antibiotic discontinuation 24 h after neonatal LOS work-up. Positive blood culture or sick appearance were selected as variables for the first node. The blood culture and sick appearance identified 64% of infants with LOS, with the remaining 36% identified by CRP and NLR. The AUC for our decision tree was 0.94 (95% CI, 0.90–0.98). We assessed our decision tree performance by use of a temporal validation cohort with an AUC of 0.92 (95% CI, 0.84–0.99). Comparisons between our decision tree to previously identified sepsis markers in both the development and temporal validation cohorts are presented in Table 3. The AUC using our decision tree was higher than previously reported sepsis markers.

**Table 3:** Comparing performance of clinical and laboratory predictors of neonatal late-onset sepsis in development and validation cohorts.

	***P*-value**	AUC^*^ (95% CI^**^)	**Sensitivity (%)**	**Specificity (%)**	PPV^***^ (%)	**NPV^**#**^ (%)**
**Development cohort (** ***n*** **=** **105)**
Decision tree analysis	<0.001	0.94 (0.90-0.98)	100	75.0	64.7	100
Positive blood culture at 24 h	0.003	0.68 (0.56-0.80)	36.4	100	100.0	77.4
Sick appearance^##^	0.001	0.70 (0.58-0.82)	42.4	97.2	87.5	78.7
NLR^###^ > 1.5	<0.001	0.88 (0.82-0.95)	100.0	76.4	66.0	100.0
CRP^§^> 0.75 mg/dl	<0.001	0.89 (0.81-0.96)	90.9	86.1	75.0	95.4
NLR > 1.5 or CRP > 0.75 mg/dl	<0.001	0.86 (0.79-0.93)	100.0	75.00	64.7	100.0
Abnormal neutrophil count^§§^	<0.001	0.75 (0.66-0.85)	90.9	59.2	50.9	93.5
**Validation cohort (** ***n*** **=** **60)**
Decision tree analysis	<0.001	0.92 (0.84-0.99)	100	52.5	52.3	100
Positive blood culture at 24 h	0.005	0.73 (0.57-0.88)	45.0	100	100	78.4
Sick appearance	0.002	0.75 (0.61-0.89)	65.0	85.4	68.4	83.3
NLR > 1.5	<0.001	0.80 (0.68-0.92)	90.0	70.0	60.0	93.3
CRP > 0.75 mg/dl	<0.001	0.84 (0.73-0.94)	95.0	72.5	63.3	96.7
NLR > 1.5 or CRP > 0.75 mg/dl	<0.001	0.74 (0.61-0.86)	95.0	52.5	50.0	95.5
Abnormal neutrophil count	0.002	0.75 (0.62-0.88)	90.0	60.0	52.9	92.3

* *AUC, area under the curve;*

** *CI, confidence interval;*

*** *PPV, positive predictive values;*

# *NPV, negative predictive value;*

## *Calculated for healthy or equivocal vs. sick appearing infants within 24 h from work-up;*

### *NLR, Neutrophil to lymphocyte ratio; ^§^CRP, C-reactive protein; ^§§^neutrophil count <1,800/mm^3^ or > 5,400/mm^3^*.

## Discussion

LOS is a common issue in any NICU, however despite implementation of antibiotic stewardship there is limited data on early antibiotic discontinuation in infants suspected of LOS and even less data when the time-frame for antibiotic discontinuation is 24 h after initiation. Our aim was to create a method that would safely exclude LOS within 24 h after initial evaluation. Use of a decision tree model, which included blood culture status, clinical sick appearance, CRP and NLR, to stop antibiotics at 24 h during the neonatal period, helped identify all culture-positive septic newborns while enabling antibiotic discontinuation in about two-thirds of non-septic infants. Our research presents a novel approach using a decision tree to minimize antibiotic duration while maintaining a very low risk of inappropriately discontinuing antibiotics in infants with sepsis.

In 2002, Kaiser et al. (22) posed the question of whether antibiotics should be discontinued 48 h after LOS work-up. Their conclusion was that this should be the standard of care. Since their publication various attempts have been made to further decrease the duration of antibiotic therapy. The motivation for decreased antibiotic duration is obvious. Increased antibiotic use not only directly impacts the health care system by increasing NICU length of stay, health care costs and resistance to antibiotics, but also has direct long-term adverse effects on the infant such as altered gut microbiota (5, 23) and obesity (6). Early cessation of antibiotic therapy enables recovery of the normal gut microbiota (23). However, discontinuing antibiotics after 24 h entails the risk of not continuing appropriate therapy in an infant with sepsis.

Use of various sepsis markers for LOS evaluation is ubiquitous. CBC for sepsis evaluation commonly includes a leukocyte count and the immature to mature neutrophil ratio. These markers lack the adequate sensitivity needed to rule-out sepsis as single markers (15, 24, 25). A less known, but readily available marker is the NLR (16). During sepsis, neutrophil counts increase due to increased release of immature neutrophils and delayed apoptosis, whereas the lymphocyte count decreases (26). The combined effect of increased neutrophils and decreased lymphocytes is an increased NLR. Because NLR takes into account both lymphocyte and neutrophil changes, it is potentially a more accurate marker for sepsis than using only lymphocyte or neutrophil counts. To date no single marker has been found to be accurate enough for rapid identification or exclusion of sepsis within 24 h. For this reason, we used a combination of sepsis markers that are readily available in the NICU. We sought to include a simple clinical evaluation as we deemed it unlikely that any treating physician would rapidly discontinue antibiotics in any sick-appearing infant.

Use of blood cultures is the gold standard for diagnosis of sepsis. In previous studies the time to positivity of cultures varied. Two studies (27, 28) including neonatal blood cultures from early and late onset sepsis showed 24-h positivity rates of 56.1 and 77%. For LOS a 24-h positivity rate of 51.7% was reported by Abdelhamid (29). Recently, Ur Rehman Durrani et al. (30) reported a 24-h positivity rate of 71.9%. The authors suggested discontinuing antibiotic therapy for Gram negative bacteria at 24 h, however recommended continuing therapy for Gram positive bacteria for which time to positivity was often longer than 24 h. In our study the 24-h positivity rate was only about 40%. In contrast to previous studies (27–29), we calculated the real time to positivity based on the time the blood culture was obtained to the time the result was communicated to the NICU and not on the minimum theoretical time indicated by the computerized blood culture system. While a positive blood culture mandates antibiotic therapy, a negative culture at 24 h without additional support is not sufficiently conclusive for antibiotic discontinuation.

Use of the combination of CRP and CBC for discontinuation of antibiotics at 36 h after LOS work-up has previously been suggested by Beltempo et al. (31). CBC abnormalities in this study were abnormal white blood cell count, thrombocytopenia or a high immature neutrophil count. However, considering the price of inappropriate antibiotic discontinuation in a septic infant, exclusive use of laboratory markers, when below cut-off values, was not accurate enough for a categorical recommendation to discontinue antibiotics at 24 h. Thus, addition clinical markers need to be used for decision making at 24 h. Our decision tree model used a similar approach but incorporated clinical evaluation, blood culture status as well as maximal CRP and NLR values at 24 h after work-up. Because both sick appearance and positive cultures clinical circumstances in which early antibiotic discontinuation would not be considered by most if not all treating physicians, we chose to group them together at the beginning of our decision tree.

The study cohort included a relatively large cohort of infants, obtained from a single large NICU. The study findings were validated using a temporal validation cohort. Splitting the cohort enables a more accurate evaluation of the suggested model, assists in avoiding overfitting of the model and is the preferred statistical approach (21). During the study period no changes were made in antibiotic policy. Infants with congenital anomalies or infants with prior early-onset sepsis that were possibly at increased risk for sepsis were excluded. The study was performed in a tertiary referral center accounting for the relatively high number of major malformations in our cohort. The study used a strict definition of sepsis based only on blood cultures. Sepsis markers readily available in any NICU were used. Despite the study strengths, some limitations should be acknowledged. The study's retrospective design has known limitations. Clinical evaluation is subjective, may be abnormal due to disease states other than LOS and was not always assigned at time of LOS. The limitations of clinical evaluation were mitigated by use of a standardized scoring sheet and by its blind performance by 2 neonatologists. Although the study was of sufficient statistical power to identify multiple risk factors for LOS, a larger multicenter cohort would strengthen the generalizability and validity of the study results, as would inclusion of more full-term infants.

In conclusion, the study presents a simple approach, feasible in any NICU, enabling identification of infants at low-risk of LOS, only 24 h after initial evaluation. In non-sick appearing infants with a negative blood culture at 24 h and normal laboratory values, sepsis is highly unlikely and antibiotics may be discontinued. The suggested approach can reduce the duration of antibiotic therapy in low-risk infants without inappropriately discontinuing antibiotics in septic infants. Additional research is needed to confirm our study results.

## Data Availability Statement

The datasets presented in this article are not readily available because research is still being conducted, however, data will be made available for collaboration purposes. Requests to access the datasets should be directed to Gil Klinger, gilkl@tauex.tau.ac.il.

## Ethics Statement

The studies involving human participants were reviewed and approved by Rabin Medical Center Research Ethics Board, Rabin Medical Center, Petah Tikva, Israel. Written informed consent from the participants' legal guardian/next of kin was not required to participate in this study in accordance with the national legislation and the institutional requirements.

## Author's Note

The study was presented in part in the annual Pediatric Academic Society 2019 meeting.

## Author Contributions

OG and GK contributed to leading role in all study aspects including: conception and design of study, data acquisition, analysis and interpretation, writing of manuscript, and final approval of the submitted manuscript. NA and HB-Z contributed to study conception and design, data acquisition, critical revision of manuscript, and approved the submitted manuscript. NS contributed to data acquisition and critical revision of manuscript and approved the submitted manuscript. RB contributed to study conception and design, data acquisition and interpretation, and critical revision of manuscript and approved the submitted manuscript. GC contributed to study conception and design, data analysis and interpretation, critical revision of manuscript, and approved the submitted manuscript. OS contributed to study conception and design, data interpretation, critical revision of manuscript, and approved the submitted manuscript. All authors agree to be accountable for all aspects of the work.

## Conflict of Interest

The authors declare that the research was conducted in the absence of any commercial or financial relationships that could be construed as a potential conflict of interest.

## Publisher's Note

All claims expressed in this article are solely those of the authors and do not necessarily represent those of their affiliated organizations, or those of the publisher, the editors and the reviewers. Any product that may be evaluated in this article, or claim that may be made by its manufacturer, is not guaranteed or endorsed by the publisher.

## Abbreviations

NICU: Neonatal Intensive Care Unit
LOS: Late-onset sepsis
CBC: complete blood count
CRP: C-reactive protein
NLR: Neutrophil-to-Lymphocyte ratio
CI: Confidence interval
OR: Odds ratio
SD: standard deviation
PPV: Positive predictive value
NPV: negative predictive value.

## References

[1] Shane AL Sánchez PJ Stoll BJ. Neonatal sepsis. Lancet. (2017) 390:1770–80. 10.1016/S0140-6736(17)31002-4 28434651

[2] Dong Y Speer CP. Late-onset neonatal sepsis: recent developments. Arch Dis Child Fetal Neonatal Ed. (2015) 100:F257–63. 10.1136/archdischild-2014-306213 25425653 PMC4413803

[3] Qazi SA Stoll BJ. Neonatal sepsis: a major global public health challenge. Pediatr Infect Dis J. (2009) 28:S1–2. 10.1097/INF.0b013e31819587a9 19106756

[4] Kuppala VS Meinzen-Derr J Morrow AL Schibler KR. Prolonged initial empirical antibiotic treatment is associated with adverse outcomes in premature infants. J Pediatr. (2011) 159:720–5. 10.1016/j.jpeds.2011.05.033 21784435 PMC3193552

[5] Gasparrini AJ Wang B Sun X Kennedy EA Hernandez-Leyva A Ndao IM. Persistent metagenomic signatures of early-life hospitalization and antibiotic treatment in the infant gut microbiota and resistome. Nat Microbiol. (2019) 4:2285–97. 10.1038/s41564-019-0550-2 31501537 PMC6879825

[6] Saari A Virta LJ Sankilampi U Dunkel L Saxen H. Antibiotic exposure in infancy and risk of being overweight in the first 24 months of life. Pediatrics. (2015) 135:617–26. 10.1542/peds.2014-3407 25825533

[7] Teo SM Mok D Pham K Kusel M Serralha M Troy N. The infant nasopharyngeal microbiome impacts severity of lower respiratory infection and risk of asthma development. Cell Host Microbe. (2015) 17:704–15. 10.1016/j.chom.2015.03.008 25865368 PMC4433433

[8] Verstraete EH Blot K Mahieu L Vogelaers D Blot S. Prediction models for neonatal health care-associated sepsis: a meta-analysis. Pediatrics. (2015) 135:e1002–14. 10.1542/peds.2014-3226 25755236

[9] Sharma D Farahbakhsh N. Biomarkers for diagnosis of neonatal sepsis: a literature review. J Matern Fetal Neonatal Med. (2018) 31:1646–59. 10.1080/14767058.2017.1322060 28427289

[10] Brown JVE Meader N Cleminson J McGuire W. C-reactive protein for diagnosing late-onset infection in newborn infants. Cochrane Database Syst Rev. (2019) 1:CD012126. 10.1002/14651858.CD012126.pub2 30640979 PMC6373636

[11] Goldberg O Amitai N Chodick G Bromiker R Scheuerman O Ben-Zvi H. Can we improve early identification of neonatal late-onset sepsis? A validated prediction model. J Perinatol. (2020) 40:1315–22. 10.1038/s41372-020-0649-6 32203177

[12] Escobar GJ Puopolo KM Wi S Turk BJ Kuzniewicz MW Walsh EM. Stratification of risk of early-onset sepsis in newborns ≥ 34 weeks' gestation. Pediatrics. (2014) 133:30–6. 10.1542/peds.2013-1689 24366992 PMC4079292

[13] Kuzniewicz MW Puopolo KM Fischer A Walsh EM Li S Newman TB. A quantitative, risk-based approach to the management of neonatal early-onset sepsis. JAMA Pediatr. (2017) 171:365–71. 10.1001/jamapediatrics.2016.4678 28241253

[14] Walker SAN Cormier M Elligsen M Choudhury J Rolnitsky A Findlater C. Development, evaluation and validation of a screening tool for late onset bacteremia in neonates - a pilot study. BMC Pediatr. (2019) 19:253. 10.1186/s12887-019-1633-1 31340780 PMC6651932

[15] Hornik CP Benjamin DK Becker KC Benjamin DK Jr Li J Clark RH. Use of the complete blood cell count in late-onset neonatal sepsis. Pediatr Infect Dis J. (2012) 31:803–7. 10.1097/INF.0b013e31825691e4 22531232 PMC3399981

[16] Alkan Ozdemir S Arun Ozer E Ilhan O Sutcuoglu S. Can neutrophil to lymphocyte ratio predict late-onset sepsis in preterm infants? J Clin Lab Anal. (2018) 32:e22338. 10.1002/jcla.22338 29055117 PMC6817131

[17] Christensen RD Baer VL Gordon PV Henry E Whitaker C Andres RI. Reference ranges for lymphocyte counts of neonates: associations between abnormal counts and outcomes. Pediatrics. (2012) 129:e1165–72. 10.1542/peds.2011-2661 22508916

[18] Klinger G Chin CN Beyene J Perlman M. Predicting the outcome of neonatal bacterial meningitis. Pediatrics. (2000) 106:477–82. 10.1542/peds.106.3.477 10969090

[19] Zhang H Singer B. Recursive Partitioning in the Health Sciences. 1st ed, New York, NY: Springer-Verlag (1999). p. 7–19 10.1007/978-1-4757-3027-2_2

[20] Kim JM Kim J. Prediction model for the differential diagnosis of Kawasaki Disease and acute cervical lymphadenitis in patients initially presenting with fever and cervical lymphadenitis. J Pediatr. (2020) 225:30–6. 10.1016/j.jpeds.2020.05.031 32450069

[21] Moons KGM Altman DG Reitsma JB Ioannidis JPA Macaskill P Steyerberg EW. Transparent reporting of a multivariable prediction model for individual prognosis or diagnosis (TRIPOD): explanation and elaboration. Ann Intern Med. (2015) 162:W1–W73. 10.7326/M14-0698 25560730

[22] Kaiser JR Cassat JE Lewno MJ. Should antibiotics be discontinued at 48 hours for negative late-onset sepsis evaluations in the neonatal intensive care unit? J Perinatol. (2002) 22:445–7. 10.1038/sj.jp.7210764 12168120

[23] Zwittink RD Renes IB van Lingen RA van Zoeren-Grobben D Konstanti P Norbruis OF. Association between duration of intravenous antibiotic administration and early-life microbiota development in late-preterm infants. Eur J Clin Microbiol Infect Dis. (2018) 37:475–83. 10.1007/s10096-018-3193-y 29368074 PMC5816780

[24] Makkar M Gupta C Pathak R Garg S Mahajan NC. Performance evaluation of hematologic scoring system in early diagnosis of neonatal sepsis. J Clin Neonatol. (2013) 2:25–9. 10.4103/2249-4847.109243 24027741 PMC3761960

[25] Rosenfeld CR Shafer G Scheid LM Brown LS. Screening and serial neutrophil counts do not contribute to the recognition or diagnosis of late-onset neonatal sepsis. J Pediatr. (2019) 205:105–11.e2. 10.1016/j.jpeds.2018.09.024 30318373

[26] Delano MJ Ward PA. Sepsis-induced immune dysfunction: can immune therapies reduce mortality? J Clin Invest. (2016) 126:23–31 10.1172/JCI82224 26727230 PMC4701539

[27] Garcia-Prats JA Cooper TR Schneider VF Stager CE Hansen TN. Rapid detection of microorganisms in blood cultures of newborn infants utilizing an automated blood culture system. Pediatrics. (2000) 105 (3 Pt 1):523–7. 10.1542/peds.105.3.523 10699103

[28] Guerti K Devos H Ieven MM Mahieu LM. Time to positivity of neonatal blood cultures: fast and furious? J Med Microbiol. (2011) 60 (Pt 4):446–53. 10.1099/jmm.0.020651-0 21163823

[29] Abdelhamid SM. Time to positivity and antibiotic sensitivity of neonatal blood cultures. J Glob Infect Dis. (2017) 9:102–7. 10.4103/jgid.jgid_1_17 28878521 PMC5572193

[30] Ur Rehman Durrani N Rochow N Alghamdi J Pelc A Fusch C Dutta S. Minimum duration of antibiotic treatment based on blood culture in rule out neonatal sepsis. Pediatr Infect Dis J. (2019) 38:528–32. 10.1097/INF.0000000000002182 30169482

[31] Beltempo M Viel-Thériault I Thibeault R Julien AS Piedboeuf B. C-reactive protein for late-onset sepsis diagnosis in very low birth weight infants. BMC Pediatr. (2018) 18:16. 10.1186/s12887-018-1002-5 29382319 PMC5791164

