# Supplementary material for: Antibiotic Discontinuation 24 h After Neonatal Late-Onset Sepsis Work-Up—A Validated Decision Tree Model
Source: Front Pediatr. 2021 Aug 19;9:693882. doi: 10.3389/fped.2021.693882 (PMC8417412; doi:10.3389/fped.2021.693882)
Supplement: Supplementary file 1 [file Table_1.DOCX]

**Appendix 1- Hierarchical classification of clinical signs**

| **Clinical presentation** | **Description** |
| --- | --- |
| **Healthy** | *Normal physical exam*, no abnormal symptoms |
| **Equivocal** | *One abnormality* (tachypnea, dyspnea, pallor, skin mottling, tachycardia, abnormal temperature, abdominal distension, irritability or hypotension) |
| **Sick appearing** | *Sick appearing* (as described by examining physician at time of sepsis evaluation per physical exam), or  *Hemodynamic instability* (hypotension requiring treatment by fluids or medications), or  *Respiratory instability* (any initiation of respiratory support, any change from non-invasive to invasive ventilation or multiple apneic or desaturation episodes), or  *Multiple abnormal symptoms* |
